# The impact of the Schmetterling NBI Program on selective eating behavior: evaluation of creative therapeutic interventions across three families of children with autism spectrum disorder

**DOI:** 10.1186/s40337-026-01597-8

**Published:** 2026-05-02

**Authors:** Sofya A. H. Bajaa, Basel Allozy, Martin Hautzinger, Ben Godde, Thomas Lang, Ahmed A. Karim

**Affiliations:** 1 Department of Psychology School of Business Social and Decision Science, Constructor University, Campus Ring 1, 28759 Bremen, Germany; 2 Practice for Child and Adolescent Psychiatry, Berlin, Germany; 3 Department of Clinical Psychology, University of Tübingen, Tübingen, Germany; 4 Tübingen Academy for Child and Adolescent Psychotherapy (TAKT), Tübingen, Germany; 5 Department of Psychiatry and Psychotherapy, University of Tübingen, Tübingen, Germany

**Keywords:** Autism spectrum disorder (ASD), Selective eating, Schmetterling NBI Program, Nutritional behavior intervention (NBI), Food refusal, Sensory processing, Evidence-based practices, Creative therapeutic interventions with children

## Abstract

Children with autism spectrum disorder (ASD) frequently exhibit selective eating behaviors characterized by food refusal and limited dietary variety, which can lead to nutritional deficiencies and impaired family functioning. This study evaluated the effectiveness of the Schmetterling Nutritional Behavior Intervention (NBI) Program, a creative behavioral intervention integrating established behavioral strategies with innovative components, including imitation chaining, shaping, and therapist-guided exoskeleton modeling. Three children (YW, RA, and JK) diagnosed with autism spectrum disorder (ASD) participated in a single-case experimental design. The study employed the Childhood Autism Rating Scale, 2nd Edition (CARS-2) assessing autism symptom severity, while the Child Eating Behavior Questionnaire (CEBQ) measured changes in eating behavior. Substantial increases in food acceptance were observed across all participants, with the highest improvements in food responsiveness, enjoyment of food, and food fussiness. Tau-U analysis revealed large and significant intervention effects for both therapist-implemented and parent-implemented sessions. Although CARS-2 scores remained within the ‘severe’ classification range, notable percentage reductions suggested clinically relevant improvements in core autism symptoms. These findings support the Schmetterling NBI Program as an individualized, evidence-based approach to enhancing dietary diversity and reducing maladaptive feeding behaviors in children with ASD.

## Plain Language Abstract

Children with autism spectrum disorder (ASD) commonly demonstrate selective eating patterns, including food refusal and restricted dietary repertoire, which may contribute to nutritional deficits and compromised family dynamics. This investigation examined the efficacy of the Schmetterling Nutritional Behavior Intervention (NBI) Program, a novel behavioral intervention that synthesizes established behavioral methodologies with innovative components such as imitation, shaping procedures, and therapist-guided training. Utilizing a single-case experimental design, three children diagnosed with ASD (YW, RA, and JK) were assessed with the Childhood Autism Rating Scale, Second Edition (CARS-2) to evaluate autism symptom severity, and the Child Eating Behavior Questionnaire (CEBQ) to measure alterations in feeding behavior. Results indicated substantial improvements in food acceptance across all participants, with pronounced enhancements in food responsiveness, enjoyment of food, and reductions in food fussiness. Tau-U statistical analysis demonstrated large and significant intervention effects for both therapist-administered and parent-implemented sessions. Although CARS-2 scores remained within the severe classification range, notable percentage reductions indicated clinically meaningful improvements in core autism symptomatology. These findings support the Schmetterling NBI Program as an individualized, evidence-based intervention for promoting dietary diversity and reducing maladaptive feeding behavior among children with ASD. Replicating our preliminary findings in a larger sample and finding evidence-based tailored interventions for children with ASD pose important challenges for future research.

## Introduction

Children with autism spectrum disorder (ASD) often exhibit selective eating behaviors, characterized by a strong aversion to certain foods and limited dietary options [15]. This habit coupled with other challenging mealtime behaviors such as spitting, hair pulling, or throwing food on the floor can severely impact the well-being, development, and social participation of individuals with ASD [8, 25]. The development of creative, tailored therapeutic interventions, especially for young children with ASD, is therefore a vital responsibility for clinical practice. In this regard, pathologic food behaviors patterns in children with ASD tend to favor high-calorie, low-nutrient foods, such as sugary drinks and processed snacks [62]. In contrast, their intake of fruits, vegetables, and other nutrient-dense foods are likely to remain low (Biesalski et al., 2015) [9]. Compounding these concerns, gastrointestinal symptoms are reported in approximately 46% of children with ASD, a prevalence significantly higher than that observed in their neurotypical peers [4, 49].

In Essence, feeding difficulties affect nearly one in four children in general pediatric populations, the markedly higher rates in children with developmental disorders which emphasize the clinical significance of addressing feeding issues in this population [12, 37]. As a result of such selective eating behaviors, children with ASD often suffer from nutritional deficiencies, impaired growth, and social challenges that compromise their overall well-being [43]. These risks highlight the urgent need for a tailored intervention targeting feeding behaviors [40]. Although selective eating is not a formal diagnostic criterion, it is widely considered a prominent behavioral feature in people with ASD [5]. Importantly, selective eating is not exclusive to children with ASD, it is also commonly reported in typically developing children, particularly between the ages of two and six years, though generally with less persistence and severity [51].

Thus, children with ASD frequently experience gastrointestinal disturbances that contribute to feeding difficulties, including chronic constipation, abdominal pain (with or without diarrhoea), and encopresis [33, 35, 48]. Many also present with motor and oral-motor coordination deficits, impairing critical feeding skills such as chewing, swallowing, and using utensils [58]. Beyond physiological barriers, psychological factors further complicate the eating behaviors of children with ASD. Notably, the high prevalence of anxiety disorders in individuals with ASD often leads to food-related phobias including neophobia, a pronounced fear of trying unfamiliar foods which amplifies resistance to dietary variety and exacerbates feeding challenges [34].

While selective eating can result from medical causes, such as gastrointestinal disorders, sensory processing difficulties, or oral-motor impairments that may respond to medical or therapeutic intervention, it is more often the product of complex biopsychosocial interactions. These behaviors often reflect an interplay between psychological rigidity, sensory sensitivities, and neurological predispositions [34].

Hemdi [22] identified behavioral problems during mealtimes as one of the most significant challenges associated with ASD in children. To explore how cultural and environmental contexts affect these challenges, Adams et al. [1] conducted a qualitative study in South Africa involving mothers of children with ASD. Their findings revealed that feeding difficulties often increase parental stress levels, mostly affecting sibling dynamics, intensify the caregiving burden on mothers, and increase the financial strain incurred by specialized diets or interventions [41].

Feeding issues are now widely acknowledged as a core clinical concern in children with ASD, leading to increased demand for precise diagnostic and therapeutic approaches tailored to the unique behavioral and sensory profiles of children with ASD [6, 24, 38, 47]. Despite the proliferation of intervention models, relatively few are supported by robust empirical evidence. For example, while the Sequential Oral Sensory (SOS) approach remains popular in practice, it lacks comprehensive scientific validation [34].

Behavioral feeding interventions, integrating structured programs such as EAT-UP, have demonstrated promising outcomes in reducing maladaptive mealtime behaviors and increasing food acceptance among children with ASD [11]. Despite these encouraging developments, many caregivers of autistic children continue to report persistent feeding challenges [20]. Even when physiological issues such as gastrointestinal discomfort are adequately addressed, food refusal often persists, driven by underlying anxiety, rigid behavioral patterns, and stress-induced avoidance [3]. In such cases, cognitive-behavioral strategies have shown growing efficacy in reducing food-related anxiety and promoting greater behavioral flexibility, highlighting the need for integrated interventions that account for both the biological and psychological dimensions of selective eating in children with ASD [55].

Nevertheless, significant research gaps persist in the understanding and treatment of selective eating. Many feeding patterns remain undertreated or poorly conceptualized, exposing the urgent need for individualized, evidence-based interventions that fully account for the complexity of each person [34, 54]. Furthermore, a major limitation in current research is its reliance on controlled clinical environments, which often fail to capture the dynamic, understanding realities of family mealtimes: real-world factors, such as parental behaviors, cultural practices, and child-specific traits, are frequently overlooked. Intervention programs like BUFFET or standardized meal plans may offer structured strategies, yet they often neglect the individual differences that influence feeding behaviors in naturalistic settings.

The development of comprehensive therapeutic interventions, especially for young children with ASD, remains a key challenge for clinical practice. Recent iterations of the Schmetterling NBI Program incorporate sensorimotor-behavioral and psychophysiological methods specifically designed for implementation in naturalistic family and social environments. The program comprises multiple specialized modules, each targeting distinct challenges frequently observed in children with ASD, arranged as a therapeutic structure that integrates nutritional counselling and behavioral techniques to reduce food selectivity, improve dietary intake, and enhance overall health outcomes [26].

The intervention incorporates a range of evidence-based behavioral components to address the complex feeding difficulties frequently observed in children with ASD. Three core strategies are central to the Schmetterling NBI Program escape extinction, differential reinforcement, and non-contingent reinforcement each backed by a strong empirical foundation. Escape extinction is pivotal in reducing food refusal, preventing avoidance behavior in response to non-preferred foods. For example, frequent attempts to leave the table are a major barrier to mealtime engagement but are successfully mitigated through this method [56]. Meanwhile, differential reinforcement is applied to encourage positive mealtime behaviors, such as accepting new foods, along with reducing reinforcement for inappropriate responses. This approach is instrumental in shaping improved tolerance of nutrient-rich foods [46]. Complementing these strategies, non-contingent reinforcement is used to provide the children with their preferred stimuli (e.g., access to toys or verbal praise) independent of specific behaviors, thereby decreasing disruptive behaviors and creating a more supportive mealtime environment [27].

In addition to the core strategies, the intervention includes several supplementary components, such as monitoring body mass index (BMI), as well as shaping, chaining, and stimulus control, grounded in behavioral theory and varying degrees of empirical evidence. These elements are important adjuncts to the program and warrant careful implementation and further validation. Shaping and chaining are used to deconstruct the complex process of food acceptance into smaller, incremental steps, proving especially beneficial for children who exhibited severe food aversions and required structured, step-by-step guidance [16].

The inclusion of antecedent exercise is based on the theoretical premise that physical activity may reduce repetitive behaviors and increase readiness to engage in feeding. However, its specific efficacy within feeding interventions has not yet been empirically validated. Similarly, the exoskeleton approach, where the therapist physically guides the child’s movements during feeding, is conceptually innovative but lacks clinical testing. Likewise, the use of digital imitation prompts to stimulate eating behaviors, though promising in theory, remains unsubstantiated in applied feeding contexts as yet.

Existing interventions largely prioritize therapist-led strategies implemented within controlled clinical settings, and thus often overlook critical factors such as environmental diversity, cultural context, and family dynamics all of which play a substantial role in shaping the behaviors of people with ASD [18]. This narrow focus limits the validity and generalizability of such approaches. Furthermore, the high cost and limited accessibility of combined behavioral and pharmacological treatments intensify the need for feasible, individualized interventions that are tailored not only to the severity of symptoms, but also to the contextual realities of each child and family [14, 57] .

In response to the ongoing need for parent-inclusive interventions, Johnson et al. [27] introduced a structured behavioral training model known as Parent Training for Feeding (PT-F), specifically designed to address feeding difficulties in children with ASD. The program focused on empowering parents as direct intervention agents and aimed to assess its feasibility and preliminary efficacy. Over a 16-week trial, 14 children aged two to seven years along with their therapists and caregivers participated in up to nine individualized sessions covering behavioral regulation, nutritional counselling, and structured mealtime routines. The findings demonstrated strong adherence to the protocol, high treatment integrity, and therapist satisfaction. Most importantly, the intervention yielded notable improvements in feeding behaviors, reduced disruptive mealtime conduct, and lower stress levels for both caregivers and therapists, reinforcing the value of parent-mediated, behaviorally grounded feeding interventions in autism care.

The Schmetterling Program also includes parents as active co-therapists, who receive structured training in the program’s core behavioral strategies to ensure consistent implementation at home. As primary caregivers capable of consistently applying behavioral strategies within the structure of daily routines, the children’s parents play an important and active role in the implementation of the Schmetterling assessments NBI Program. To ensure familiarity with the program and competence in enacting it, they receive structured training by a trained therapist through a standardized curriculum encompassing the core principles of behavioral feeding interventions, including behavioral management, feeding skill acquisition, and consistency in mealtime routines. In line with recent literature on parent-mediated feeding interventions [7], the training is intensive and iterative, incorporating rehearsal, feedback, and performance monitoring. This approach positions the parents as first-order change agents in the intervention, acknowledging their tacit knowledge, daily presence, and lived experience as foundational assets in shaping meaningful therapeutic outcomes.

The evolution of the Schmetterling NBI Program reflects its growing potential as a holistic, individualized intervention that promotes nutritional diversity, adaptive mealtime behaviors, and improved quality of life for children with ASD and their families. Notably, the translation of efficacious feeding interventions into routine clinical practice remains limited by health-system barriers such as inadequate therapist training, high costs, and lack of reimbursement structures [14, 57]. The Schmetterling NBI Program was designed with these implementation considerations in mind, prioritizing flexibility, individualization, and caregiver involvement to enhance its translational potential. With the current study, conducted in Berlin, we aimed to evaluate the impact of the Schmetterling NBI Program on selective eating behaviors in children diagnosed with ASD. A single-case experimental design was employed and replicated across three children, with interventions implemented in a practice for Child and Adolescent Psychiatry. While we expected that therapist-led sessions would show stronger initial effects due to the therapist’s expertise, parent-led sessions would demonstrate successful skill transfer and generalization.

## Methods

### Participants

This study had three participants (RA, YW, and JK) each of whom had been diagnosed with ASD before reaching the age of three, according to the ICD-10 criteria. The children came from culturally and socioeconomically diverse backgrounds, with a view to supporting the program’s potential generalizability to different environments in the future.

- YW, a 4-year-old German citizen of Iraqi descent, lived with his married parents and two older siblings. His mother worked from home, and his father prepared meals. YW’s diet consisted largely of pizza, fried potatoes, and sugary beverages, with minimal intake of nutrient-rich foods.
- RA, a 6-year-old Syrian refugee, lived with his married parents and two older siblings. His father was unemployed, and his mother was a homemaker. RA demonstrated severe food aversions, primarily consuming chips, white bread, and chocolate spread, while completely refusing fruits, vegetables, and protein sources.
- JK, a 2.8-year-old Syrian refugee, lived with his married parents and one sibling. He favored dry and crispy foods, such as chips and cookies, and rejected all fruits and vegetables.

All three children exhibited entrenched selective eating behaviors, accompanied by mealtime challenges including verbal refusals, hyperactivity, and occasional self-injury which significantly disrupted both family dynamics and nutritional sufficiency.

The intervention’s behavioral, structural, and theoretical underpinnings are summarized in Table 1, offering a practical framework for implementing and assessing the current study.

**Table 1:** Tailored behavioral components of the Schmetterling NBI Program

Behavioral strategy	Description and application
1.Extinction of disruptive mealtime behaviors	Removal of reinforcement for inappropriate behaviors (e.g., maintaining the presence of the spoon despite refusal or crying) to reduce escape-maintained behaviors [31], applied in all sessions
2.Graduate exposure food	Graduate food exposure (graduated exposure therapy) for autism involves a systematic, incremental approach to increasing the acceptance of new foods, addressing sensory sensitivities and picky eating by exposing individuals to foods in a non-threatening way [30], implemented gradually in sessions 8, 9, and 10
3.Motor activation (antecedent exercise)	Structured physical activity (e.g., light jogging) followed by fine-motor tasks (e.g., drawing) to enhance readiness and reduce repetitive behaviors before feeding sessions [44], applied in sessions 11, 12, 17, 18, 26, and 27
4.Shaping and chaining with exoskeleton modelling	Shaping reinforces successive approximations toward a target behavior (e.g., food acceptance), while chaining teaches sequences of behaviors. In this program, the therapist may physically guide the child’s hand (exoskeleton modelling) to support food interaction. Verbal and non-verbal reinforcers (e.g., facial expressions and verbal praise) are provided for each successful step [42], applied in sessions 13, 14, 15, and 16
5.Imitation facilitation	Observational learning through video modelling (e.g., showing videos of cute baby animals eating) or structured imitation games (e.g., rhythmic tapping of a spoon on the table) to promote mealtime engagement and food acceptance [23], applied in sessions 19, 20, 21, 22, 23, 27, and 28
6.Non-contingent reinforcement (NCR)	Delivery of preferred stimuli (e.g., praise, toys, or access to preferred foods) independent of the child’s behaviors, thus reducing the motivation to engage in disruptive behaviors by saturating access to reinforcers, applied in sessions 24 and 25

### Setting and research design

The study employed a single-subject A-B-A design to evaluate the effectiveness of the Schmetterling NBI Program in modifying selective eating behaviors in children diagnosed with ASD. Following the intervention, a three-session withdrawal phase was implemented where parents were instructed to revert to pre-intervention mealtime routines. Data showed maintenance of gains achieved, suggesting behavioral maintenance. This design was selected due to its suitability for capturing individual-level behavioral changes, making it particularly well-aligned with the personalized and context-sensitive nature of feeding interventions. To enhance experimental control and improve internal validity, the study used an A-B-A variation, in which participants were temporarily returned to baseline conditions following the initial improvements. This approach allowed for a rigorous assessment of whether increases in food acceptance were directly attributable to the intervention itself, or to extraneous variables [36].

To enhance the internal validity and reliability of the findings, the same procedure was repeated across all three participants, thereby increasing the consistency of observed outcomes. The feeding sessions were primarily conducted in a practice for Child and Adolescent Psychiatry in Berlin, Germany. Small portions were initially offered to the participants in order to reduce anxiety and maximize the chance of food acceptance [46, 56]. Reinforcement contingencies, such as verbal praise or access to preferred activities, were employed throughout the exposure process to strengthen the participants’ engagement with the target foods. This flexible, child-centered approach allowed the parents and therapists implementing the intervention to adjust the method and the pacing of their facilitation in response to real-time behavioral cues, thus fostering a personalized and developmentally appropriate mealtime experience for each of the participants.

The frequency of target eating behaviors including acceptance, refusal, expulsion, and disruptive responses was systematically recorded through direct observation during each session. Two trained clinical assistants independently served as observers for all sessions who captured real-time behavioral data from the child, the parent, and the therapist. To prevent observer drift and ensure consistency, the observers alternated in their roles (primary vs. secondary coder) across sessions for each participant, rather than being permanently assigned to a specific child. This rigorous data collection ensured that the fidelity with which the program was being implemented was consistently monitored, and that each child’s sensory and behavioral responses could inform tailored adjustments throughout the intervention process.

The following behavioral categories were coded during each session:

- acceptance: the child placed food from the spoon into his mouth.
- mouth cleaning: the child removed food from his mouth using his hand or tongue.
- refusal: the child actively declined to put food in his mouth.
- expulsion: the child ejected food from his mouth after partial or full insertion.

Disruptive behaviors were also tracked during each session, and included a broad range of actions:

- crying: visible tears or eye moisture.
- screaming: loud, emotionally charged vocalizations.
- pushing food away: actively moving the spoon or plate away.
- self-injury: self-directed aggression (e.g., the child hitting himself).
- aggression: frustrated or resistant actions toward the feeding activity.
- attempting to leave trying to get out of the chair or unbuckle the strap.
- head turning or rotating the head away from the spoon to avoid eating.

### The Schmetterling NBI Program protocol

The Schmetterling Nutritional Behavior Intervention (NBI) Program, a multi-module integrative protocol, was systematically implemented to address selective eating behaviors through a combination of evidence-based behavioral strategies and creative therapeutic components. The intervention’s core applied behavioral techniques included escape extinction (removing reinforcement for food refusal), differential reinforcement (rewarding acceptance while ignoring refusal), and non-contingent reinforcement (providing preferred stimuli irrespective of behavior) to establish foundational mealtime engagement. These were supplemented by structured shaping and chaining to break feeding into manageable steps, antecedent exercise to enhance readiness, exoskeleton modelling for guided physical prompting, and imitation facilitation via video and play to promote observational learning. Parents were trained as co-therapists to ensure consistency, and the program was delivered in a standardized sequence across nutritional, behavioral, and sensorimotor modules, with the specific timing and application of each component detailed in Table 1.

### Baseline procedure

**Fig. 1:**
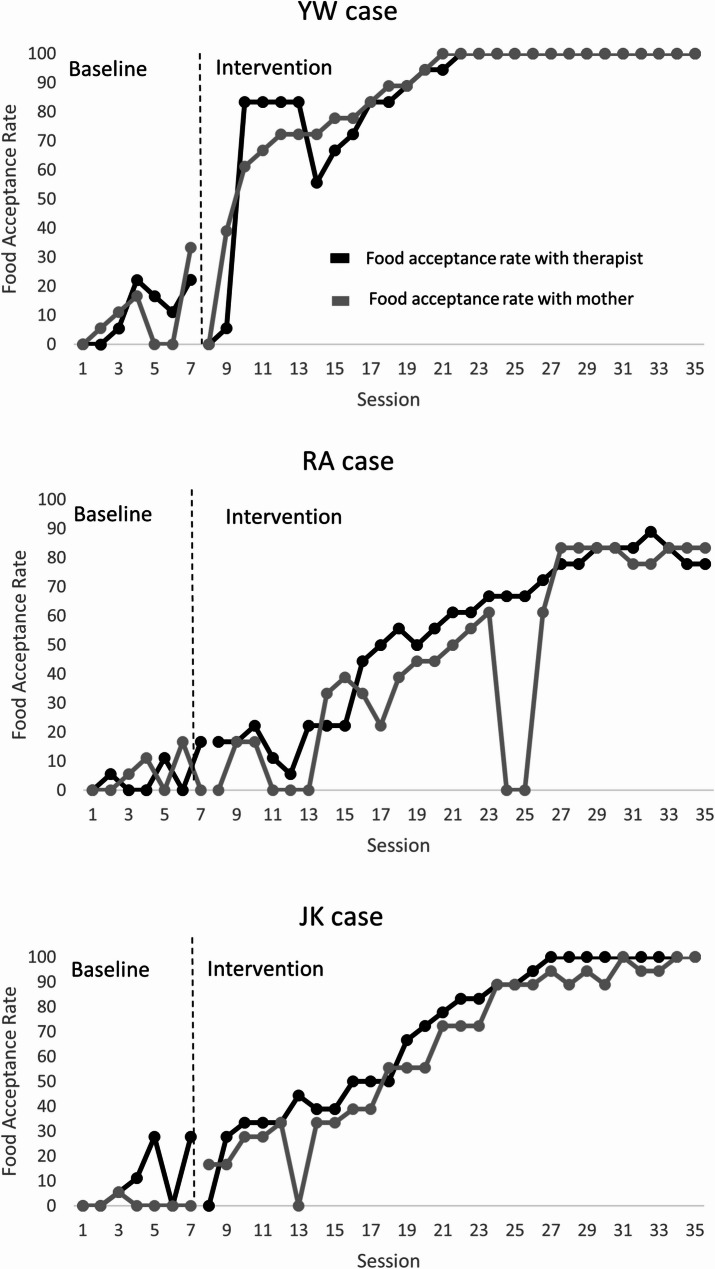
Food acceptance rates with 7 baseline and 28 intervention sessions for each participant. Each data point represents the percentage of acceptance in one 3-minute coded interval. Note: Zero values indicate no food acceptance during that session. Tau-U is a non-parametric rank-order statistic that compares the ordinal position of data points rather than their absolute values; therefore, zero values are handled robustly without requiring transformation or adjustment [45]

The baseline phase aimed to establish a reliable pre-treatment reference point by observing and recording each child’s typical mealtimes without intervention across seven structured sessions with the mothers and therapist (see Fig. 1). To ensure consistency and reliability, data collection incorporated both direct behavioral observation and standardized tools, such as the Child Eating Behavior Questionnaire (CEBQ) [66]. Additionally, the Childhood Autism Rating Scale, 2nd Edition (CARS-2; [53]), was administered to assess autism-related behavioral characteristics during the baseline procedure, in order to generate further context for interpreting each child’s feeding responses. During the baseline sessions, all responses were video recorded to allow for detailed post-session analysis. The participants were presented with suitable foods like pieces of carrot, lettuce, and apple across 18 structured attempts, with six attempts allocated to each of the three food items. The trained observer documented a range of behaviors, including food acceptance, disruptive responses, and general dietary preferences. For every attempt, acceptance of the presented item (e.g., taking, chewing, or swallowing) was coded as 1, whereas refusal or non-engagement was coded as 0, generating a systematic record of the child’s behavioral responses across the various attempts made for each food type.

### Intervention procedure and implementation

Following the baseline phase, the research team collaborated with each child’s therapist to establish individualized treatment goals and develop a tailored intervention plan. This plan was carefully aligned with the child’s developmental profile, family routines, and personal strengths. To maintain consistency across participants, the intervention adhered to a standardized structure and sequence despite its personalized elements. The Schmetterling NBI Program was delivered over 10 weeks through 28 sessions, each lasting approximately 90 min. Subsequent sessions introduced structured strategies for:

- food exposure and acceptance.
- strengthening mealtime dynamics.
- implementing the core feeding intervention.
- facilitating the adoption of new, nutrient-dense foods.

Sessions were structured into four sequential phases: a 30-minute therapist-led activity block, a 30-minute parent-led block (applying the same techniques under supervision), a 20-minute relaxation period, and a final 10-minute Standardized Feeding Assessment. During this assessment phase, the child was gently invited to sample carrot, lettuce, and apple—each presented separately and reintroduced in varied sequences across sessions to encourage food exploration and acceptance. The child was prompted to eat the food by using, for example, the phrase ‘eat the carrot’, ‘eat the apple’, or ‘eat the lettuce’, after which no further guidance was provided: no exoskeleton modelling, physical correction, or additional requests were used. Instead, sufficient time was given for the child to respond independently to the request. The prompting phrase was repeated for each food type six times, resulting in six consecutive trials per item. All environmental and procedural conditions were fully standardized, including the use of identical glass plates and the same room, table, chair, timing, and food preparation performed consistently by both the therapist and the parent. This assessment period was recorded and included two 3-minute observational windows where food acceptance trials were coded, one for the therapist and one for the parent. To evaluate generalization across implementers, the prompting during this assessment was systematically alternated: in designated sessions, the therapist delivered the verbal prompts (“eat the carrot/lettuce/apple”) [19], while in others, the parent delivered the identical prompts. Therefore, the terms “therapist-implemented” and “parent-implemented” in the results refer specifically to data from this 3-minute assessment window under the respective prompting condition, not to separate sessions.

This behavior was selected as the sole primary outcome for three reasons. First, it directly operationalizes the intervention’s central target increasing oral intake of non-preferred foods. Second, secondary behaviors (refusal, expulsion, and seven disruptive categories) exhibited insufficient variation for statistical analysis, occurring in fewer than 5% of baseline intervals across participants. Third, analyzing multiple primary outcomes would necessitate correction for multiple comparisons, substantially reducing power in this single-case design. This approach follows established feeding intervention literature where acceptance rate is the validated indicator of treatment efficacy [46, 56].

Although each therapy session lasted 90 min, the analysis was restricted to a predefined and standardized temporal window. Specifically, only the core feeding period (minutes 20 70 of each session) was considered, as this phase reflects stable feeding behavior following initial regulation and warm-up activities. Within this period, food presentation trials occurred at fixed 5-minute intervals. To ensure consistency, feasibility, and reliability in this single-case design, one standardized 3-minute video segment was extracted from each session for coding [17]. This segment began 2 min after the first food presentation to minimize reactivity and capture a representative feeding trial. The primary outcome, percentage of food acceptance, was calculated as (intervals with acceptance / 18 intervals) × 100. This temporal sampling strategy was applied uniformly across all sessions and phases.

### Secondary outcome measures

The Child Eating Behavior Questionnaire, was completed independently by the participants’ parents at home, with the baseline assessment conducted within three days of the first session and the post-intervention assessment conducted within three days after the last session. Additionally, the Childhood Autism Rating Scale, 2nd Edition (CARS-2) was administered by the therapist following the same timing protocol. The participants’ height and weight were measured by a Physician in the practice for Child and Adolescent Psychiatry at baseline and post-intervention. Additionally, the readings were recorded using a calibrated digital scale, and BMI calculated using the formula kg/m². These measures were collected in order to assess whether improvements in food acceptance corresponded with measurable changes in growth parameters.

## Results

The primary outcome, percentage of food acceptance, was measured across 7 baseline and 28 intervention sessions for each participant. During baseline, all three children demonstrated low and variable rates of food acceptance. Mean baseline acceptance was 12% (range: 0–28%) for YW, 8% (range: 0–19%) for RA, and 15% (range: 5–32%) for JK. Following introduction of the Schmetterling NBI Program, immediate increases in acceptance were observed for all participants. During the intervention phase, mean acceptance rose to 78% (YW), 85% (RA), and 91% (JK) for therapist-implemented sessions, and to 72% (YW), 76% (RA), and 88% (JK) for parent-implemented sessions. These gains were maintained during the withdrawal phase, suggesting sustained behavioral change. Figure 1 displays the session-by-session acceptance rates for each participant across all study phases.

The observer quantified these behaviors by recording the frequency of each within its assigned category, thus enabling both descriptive and inferential analysis of behavioral trends throughout the intervention period.

Across all three participants, the visual pattern corresponds closely to the three components highlighted by Parker, Vannest and Ninci [65], for interpreting single-case graphs: level, trend, and non-overlap. All three participants demonstrated immediate and sustained increases in food acceptance following intervention’s onset (see Fig. 1). Comparing therapist-implemented and parent-implemented sessions, the patterns for all three participants show that the sessions (regardless of which adult implemented the intervention during them) follow the same general intervention trajectory. However, therapist-implemented sessions tend to rise earlier and stabilize sooner, consistent with the additively stronger non-overlap noted in immediate-response conditions [45]. Parent-implemented sessions, while improving, show slightly more variability and occasional dips, producing modest within-intervention overlap [45].

Figure 1 (YW) displays the percentage of food acceptance across 7 baseline sessions and 28 intervention sessions for participant YW. Data points represent acceptance rates during standardized 3-minute observational intervals. Black circles denote therapist-implemented sessions; grey circles denote parent-implemented sessions. Baseline acceptance was low and variable (mean = 12%). Following introduction of the Schmetterling NBI Program, an immediate and sustained increase in food acceptance was observed, with mean acceptance rising to 78% for therapist-implemented sessions and 72% for parent-implemented sessions.

Figure 1 (RA) displays the percentage of food acceptance across 7 baseline sessions and 28 intervention sessions for participant RA. Data points represent acceptance rates during standardized 3-minute observational intervals. Black circles denote therapist-implemented sessions; grey circles denote parent-implemented sessions. Baseline acceptance was low and variable (mean = 8%). Following introduction of the Schmetterling NBI Program, a gradual but sustained increase in food acceptance was observed, with mean acceptance rising to 85% for therapist-implemented sessions and 76% for parent-implemented sessions.

Figure 1 (JK) displays the percentage of food acceptance across 7 baseline sessions and 28 intervention sessions for participant JK. Data points represent acceptance rates during standardized 3-minute observational intervals. Black circles denote therapist-implemented sessions; grey circles denote parent-implemented sessions. Baseline acceptance was low and variable (mean = 15%). Following introduction of the Schmetterling NBI Program, an immediate and sustained increase in food acceptance was observed, with mean acceptance rising to 91% for therapist-implemented sessions and 88% for parent-implemented sessions

The efficacy of the Schmetterling NBI Program was quantitatively assessed using Tau-U, a non-parametric single-case analysis that integrates both phase non-overlap and within-phase trend as follows.

### Baseline (phase A) trend analysis

The initial phase analysis assessed the presence of pre-existing monotonic trends that could threaten internal validity. Low, variable rates of food acceptance were recorded across all three participants. Mean baseline acceptance was 12% (range: 0–28%) for YW; 8% (range: 0–19%) for RA; and 15% (range: 5–32%) for JK. For the therapist-implemented sessions (see Table 2), all three participants (YW, RA, and JK) exhibited positive baseline Tau-U values (0.27 to 0.53), suggesting a slight tendency for food acceptance to improve prior to intervention. However, these trends were not statistically significant (all *p* > 0.13), and their 90% confidence intervals universally encompassed zero, indicating the absence of a significant trend. For the parent-implemented sessions (see Table 3), the baselines were even more stable, with very small, non-significant trends for YW and RA (Tau-U = 0.13) and a non-significant, slightly decreasing trend for JK (Tau-U = -0.20). This pattern across both implementer groups resonates with Parker et al.,’s [45] caution that while baseline trends may be visually apparent, they are often statistically unreliable, thus justifying subsequent intervention analysis.

**Table 2:** Tau-U results for the Schmetterling NBI Program (therapist-implemented sessions)

Label	TAU-U value	Standard deviation	Z-value	*P*-value	CI 85%	CI 90%
*Baseline trend*						
YW	0.533	5.323	1.503	0.133	0.022 < > 1.000	-0.05 < > 1.000
RA	0.267	5.323	0.752	0.452	-0.244 < > 0.778	-0.317 < > 0.85
JK	0.467	5.323	1.316	0.188	-0.044 < > 0.978	-0.117 < > 1.00
*Intervention trend*						
YW	0.643	53.310	4.896	0.001	0.454 < > 0.832	0.427 < > 0.859
RA	0.830	53.310	6.322	0.001	0.641 < > 1.000	0.614 < > 1.000
JK	0.857	53.310	6.528	0.001	0.668 < > 1.000	0.641 < > 1.000
*Intervention vs. baseline*						
YW	0.885	45.695	3.370	0.008	0.507 < > 1.000	0.453 < > 1.000
RA	0.942	45.695	3.590	0.003	0.564 < > 1.000	0.511 < > 1.000
JK	0.931	45.695	3.546	0.004	0.553 < > 1.000	0.499 < > 1.000
*Weighted average*						
YW + RA+JK	0.920		6.065	0.001	0.7012 < > 1.000	0.670 < > 1.00

LL CI = lower limit confidence interval; UL CI = upper limit confidence interval; all confidence intervals are at the 90% level

**Table 3:** Tau-U results for the Schmetterling NBI Program (parent-implemented sessions)

Label	TAU-U value	Standard deviation	Z-value	*P*-value	CI 85%	CI 90%
*Baseline trend*						
YW	0.133	5.3229	0.376	0.707	-0.378 < > 0.644	-0.450 < > 0.717
RA	0.133	5.3229	0.376	0.707	-0.378 < > 0.644	-0.450 < > 0.717
JK	-0.200	5.3229	-0.564	0.573	-0.711 < > 0.311	-0.784 < > 0.384
*Intervention trend*						
YW	0.692	53.310	5.271	0.001	0.503 < > 0.881	0.476 < > 0.908
RA	0.672	53.310	5.121	0.001	0.483 < > 0.861	0.456 < > 0.888
JK	0.867	53.310	6.603	0.001	0.678 < > 1.000	0.651 < > 1.000
*Intervention vs. baseline*						
YW	0.943	45.695	3.589	0.003	0.564 < > 1.000	0.511 < > 1.000
RA	0.678	45.695	2.583	0.010	0.300 < > 1.000	0.246 < > 1.000
JK	0.959	45.695	3.655	0.003	0.582 < > 1.000	0.528 < > 1.000
*Weighted average*						
YW + RA+JK	0.860		5.673	0.001	0.641 < > 1.00	0.610 < > 1.00

LL CI = lower limit confidence interval; UL CI = upper limit confidence interval; all confidence intervals are at the 90% level

### Intervention (phase B) trend analysis

For therapist-implemented sessions, mean acceptance increased to 78% (YW), 85% (RA), and 91% (JK). For parent-implemented sessions, means were 72% (YW), 76% (RA), and 88% (JK). A key strength of Tau-U is its capacity to quantify improvement *during* the intervention phase, capturing progress beyond an immediate level change. This analysis yielded strong, statistically significant monotonic improvement trends for every participant across both implementer groups (all *p* < 0.001). For therapist-implemented sessions, the Tau-U values ranged from 0.64 (YW) to 0.86 (JK), indicating that 64% to 86% of all pairwise comparisons within the phase showed improvement. Similarly, for parent-implemented sessions, the values ranged from 0.67 (RA) to 0.87 (JK). This demonstrates that the intervention was not only associated with an initial change but also fostered a sustained, positive trajectory of progress, regardless of whether it was delivered by a therapist or a parent.

### Intervention vs. baseline (A vs. B) contrast analysis

This contrast measures the immediate and overall non-overlap, or ‘dominance,’ of the intervention phase over the baseline phase. The results here are unequivocal. For the therapist-implemented sessions, the phase contrast is exceptionally strong, with Tau-U values of 0.89 (YW), 0.94 (RA), and 0.93 (JK), all of which are highly significant (*p* < 0.001). The results for the parent-implemented sessions are similarly powerful, with two participants (YW and JK) showing near-ceiling effects (Tau-U > 0.94), while RA shows a large and significant effect (Tau-U = 0.68, *p* = 0.0098). The aggregated, weighted average Tau-U scores of 0.92 (therapist-implemented) and 0.86 (parent-implemented) confirm a very large and reliable overall treatment effect.

### Effects on weight and body mass index (BMI)

Table 4 shows the changes in weight and BMI during the intervention among the three participants. The intervention was associated with increases in weight and Body Mass Index (BMI) across all three participants, all children gained weight: YW from 20.5 kg to 22.0 kg, RA from 19.0 kg to 21.0 kg, and JK from 19.0 kg to 19.5 kg. These gains corresponded to a positive shift in BMI, moving each child to a healthier growth trajectory. The most notable increase was observed for RA, whose BMI rose from 15.70 to 17.36. These changes suggest that improved dietary acceptance translated into measurable, albeit preliminary, nutritional benefits.

**Table 4:** Changes in weight and body mass index (BMI) pre- and post-intervention

Participant	Age (years)	Height pre (cm)	Height post (cm)	Weight pre (kg)	Weight post (kg)	BMI pre	BMI post
YW	4.0	109	109	20.5	22.0	18.22	18.51
RA	6.0	110	110	19.0	21.0	15.70	17.36
JK	2.8	102	105	19.0	19.5	17.71	18.27

BMI is calculated as weight (kg) / [height (m)] ²

### Effects on severity of autism symptoms

Table 5 presents the changes in the severity of autism symptoms as assessed using the Childhood Autism Rating Scale, 2nd Edition, Standard Version (CARS-2 ST). On this scale, raw scores ranging from 30 to 36 indicate mild to moderate autism, while scores between 37 and 60 correspond to severe autism. Although all three participants remained within the severe symptom category based on their mean raw scores, the post-intervention results indicate notable symptom improvement.

**Table 5:** Changes in severity of autism symptoms according to the Childhood Autism Rating Scale, 2nd Edition, Standard Version (CARS-2 ST)

Participant	Age (years)	Raw score (pre)	Raw score (post)	Percentile (pre)	Percentile (post)
YW	4.0	51.0	37.5	95%	42%
RA	6.0	49.5	38.0	92%	50%
JK	2.8	49.5	36.5	92%	42%

Based on CARS-2 ST classifications, scores ranging from 30 to 36 are classified as mild to moderate ASD, and scores ranging from 37 to 60 are classified as severe ASD

### Evaluation of child eating behaviors

**Fig. 2:**
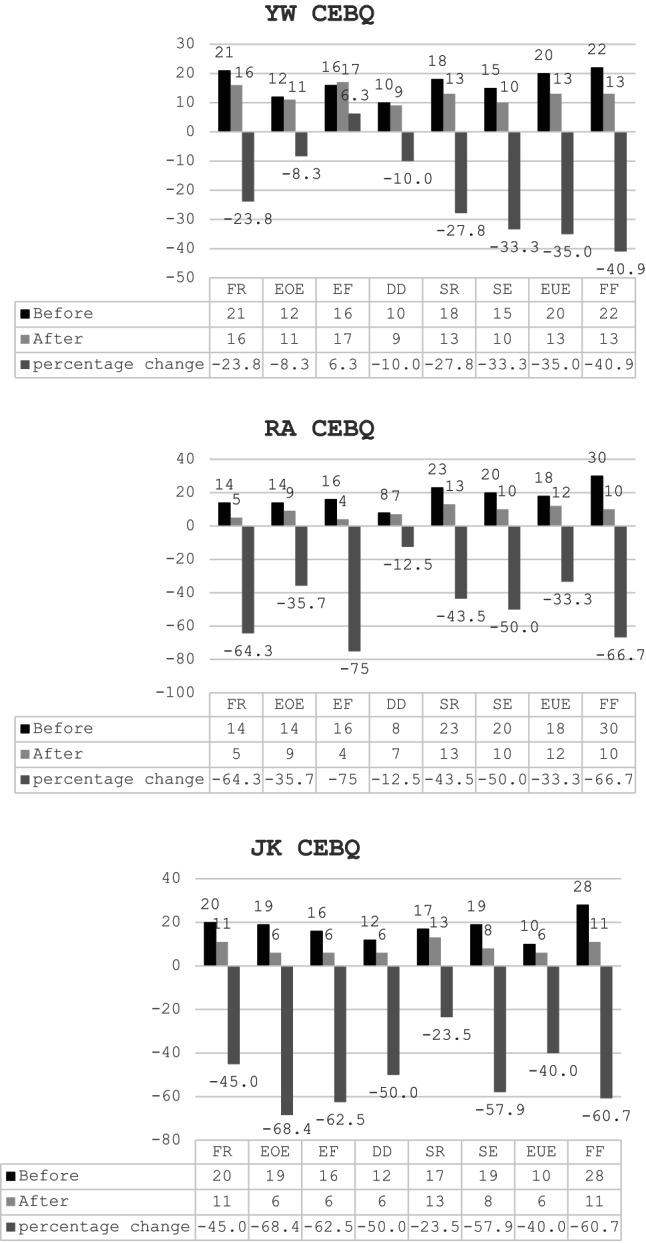
Comparison of the mean scores from the Child Eating Behavior Questionnaire conducted before and after the intervention: food responsiveness (FR), emotional over-eating (EOE), enjoyment of food (EF), desire to drink (DD), satiety responsiveness (SR), slowness in eating (SE), emotional under-eating (EUE), and food fussiness (FF)

To evaluate the impact of the intervention on the children’s eating behaviors and food-related attitudes, several subscales from the Child Eating Behavior Questionnaire (CEBQ) were assessed, namely: food responsiveness (FR), emotional over-eating (EOE), enjoyment of food (EF), desire to drink (DD), satiety responsiveness (SR), slowness in eating (SE), emotional under-eating (EUE), and food fussiness (FF). Figure 2 (YW) presents pre-intervention and post-intervention scores on the Child Eating Behavior Questionnaire (CEBQ) for participant YW. Eight subscales are displayed: food responsiveness (FR), emotional over-eating (EOE), enjoyment of food (EF), desire to drink (DD), satiety responsiveness (SR), slowness in eating (SE), emotional under-eating (EUE), and food fussiness (FF). Following the intervention, YW showed a reduction in food fussiness (-40.9%) and increases in food responsiveness and enjoyment of food, indicating improved mealtime engagement.

Figure 2 (RA) presents pre-intervention and post-intervention scores of the Child Eating Behavior Questionnaire (CEBQ) for participant RA. Eight subscales are displayed: food responsiveness (FR), emotional over-eating (EOE), enjoyment of food (EF), desire to drink (DD), satiety responsiveness (SR), slowness in eating (SE), emotional under-eating (EUE), and food fussiness (FF). Following the intervention, RA demonstrated the largest reduction in food fussiness (-66.7%) among all participants, alongside notable increases in food responsiveness and enjoyment of food.

Figure 2 (JK) presents pre-intervention and post-intervention scores of the Child Eating Behavior Questionnaire (CEBQ) for participant JK. Eight subscales are displayed: food responsiveness (FR), emotional over-eating (EOE), enjoyment of food (EF), desire to drink (DD), satiety responsiveness (SR), slowness in eating (SE), emotional under-eating (EUE), and food fussiness (FF). Following the intervention, JK showed a substantial reduction in food fussiness (-60.7%) and marked increases in food responsiveness and enjoyment of food, reflecting improved feeding behaviors.

## Discussion

The study examined the effects of the Schmetterling Nutritional Behavior Intervention (NBI) on feeding behaviors, autism symptom severity, and anthropometric indicators across three children diagnosed with ASD. Findings from the result demonstrate that the intervention produced pronounced improvements in mealtime engagement, feeding flexibility, and behavioral regulation, as reflected in substantial reductions in food fussiness and increases in food responsiveness in the CEBQ. These improvements were consistent across therapist-implemented and parent-implemented sessions. The convergence between CEBQ outcomes, reductions in CARS-2 raw scores, and maintained gains during withdrawal demonstrates broader developmental progress, rather than isolated behavioral compliance. Importantly, these improvements align with prior research showing that targeted behavioral interventions can modify feeding-related rigidity and reduce maladaptive responses associated with ASD [24, 57]. While weight and BMI changes were modest, this is expected in short-term behavioral feeding programs, particularly in children with autism since their growth trajectories can differ from those of their neurotypical peers [13, 68]. The study’s findings support the effectiveness of the Schmetterling NBI Program in enhancing both feeding behaviors and core regulatory capacities foundational to ASD symptoms.

### Changes in autism symptoms (CARS-2)

All three participants demonstrated measurable reductions in their CARS-2 raw scores. Both YW and JK had a marked improvement. The CARS-2 manual indicates that raw score decreases of two to four points in short-term interventions reflect reductions in symptom intensity, particularly improvements in behaviors regulation, adaptability, and responsivity [23]. These domains are highly relevant to feeding challenges in children with ASD and align precisely with the behavioral improvements observed in this study. Furthermore, symptom changes in early-stage interventions do not produce immediate diagnostic reclassification, rather modest reductions can be considered predictive markers of later global gains [64] . The generalization of improved regulation to mealtime contexts, combined with stable gains during withdrawal, indicates that the children internalized more adaptive behavioral and sensory patterns. These outcomes are consistent with evidence suggesting that targeted interventions focused on sensory-motor engagement can lower emotional reactivity and enhance regulatory functioning, leading to reductions in ASD symptom severity [23, 61]. Thus, the CARS-2 data suggest that autism-related symptoms improved during the intervention.

### Feeding behaviors outcomes (CEBQ)

The CEBQ findings provide preliminary evidence that the intervention produced meaningful improvements across multiple feeding-related behavioral dimensions. All participants demonstrated pronounced reductions in food fussiness, reflecting a decrease in selective eating and a greater willingness to accept foods. Food fussiness is one of the most stable indicators of feeding-related rigidity in people with ASD, strongly associated with sensory hypersensitivity and cognitive inflexibility [64, 65] . Therefore, the decreases seen in this study, especially for JK, indicate that the improvement of core ASD-linked behaviors did occur, and did not merely constitute session-specific compliance. In parallel, increases in enjoyment of food and food responsiveness demonstrate enhanced motivation to engage with food stimuli, reduced avoidance, and increased positive emotionality during meals, aligning with literature showing that behavioral feeding interventions can reduce anxiety and maladaptive responses [57]. Improvements maintained during the withdrawal phase further indicate that the children internalized behavioral changes beyond the intervention. The consistency of progress across both the therapist-implemented and parent-implemented sessions also demonstrates high generalizability, a hallmark of meaningful behavioral change. Collectively, the feeding outcomes support the conclusion that the intervention improved regulatory capacity, sensory tolerance, and flexibility dimensions strongly tied to autism symptom severity.

### Health indicators, implementation phases, and clinical implications

Given that the current study spanned a relatively brief period, the stability of BMI should be interpreted as a neutral outcome rather than a negative one. Importantly, the three children demonstrated increased dietary flexibility, which is the required precursor for long-term nutritional change [59]. The Tau-U results further reinforce the robustness of behavioral gains by indicating high non-overlap between the baseline and intervention phases, consistent with strong treatment effect magnitudes. The successful replication of gains during parent-implemented sessions and their maintenance during the withdrawal phase demonstrate that the intervention supports sustainable and transferable behavioral improvements. High social validity scores confirm that the participants’ parents perceived the program to be practical, effective, and beneficial an essential factor for real-world implementation [52]. Taken together, these findings affirm that the intervention positively impacted both ASD-related symptoms and feeding behaviors, while establishing the foundational conditions necessary for future improvements in health indicators.

### Limitations of the study

Several limitations must be acknowledged. Firstly, the sample size was limited to only three participants, which significantly restricts the generalizability of the findings to broader populations of children with ASD [21]. Although single-case experimental designs like this one are valuable for examining individual responses to intervention, they inherently limit statistical power and external validity [36, 60]. While the staggered introduction of intervention phases enhances internal validity, the lack of randomization remains a constraint. Secondly, the study relied heavily on caregiver-reported measures, such as the Children’s Eating Behavior Questionnaire, which may introduce bias due to subjective interpretation or social desirability effects [28] [2]. Likewise, the developmental range of our participants (2.8 to 6 years) introduces potential variability in intervention response that warrants careful consideration. Although a recent large-scale mega-analysis by Mandelli et al., [39] found that earlier intervention start was associated with improved developmental outcomes across multiple domains, the literature on age as a predictor remains complex. A comprehensive meta-analysis by Chetcuti et al. [10], synthesizing 95 studies encompassing 6,780 children, reported that child age at intervention onset did not significantly predict the strength of post-intervention outcomes across behavioral intervention approaches. However, this null finding may reflect the heterogeneity of outcome measures and intervention types rather than the absence of true developmental effects.

Indeed, feeding-specific developmental trajectories are well-documented. Al-Beltagi [2] describes distinct normative feeding stages, whereby children aged 2–3 years exhibit emerging chewing skills and utensil use. Whereas by 4–5 years, typically developing children demonstrate expanded dietary variety and improved social eating behavior , these milestones may be differentially delayed or disrupted in autistic children. In this regard, Klinger et al. [32] note that while age and IQ have been widely debated as predictors, differing results emerge across treatment modalities, suggesting that intervention type may interact with developmental stage. Given that our participants spanned multiple developmental feeding stages, future studies should recruit larger, developmentally stratified cohorts to examine age as a potential moderator of feeding-specific intervention outcomes. Although child outcomes were the primary focus, the magnitude of improvements observed suggests potential secondary benefits for parental and family wellbeing. Mealtime difficulties are known to elevate caregiver stress and disrupt family functioning [13, 29, 63]. Future research should therefore include validated measures of parental stress, caregiver burden, and family quality of life to comprehensively evaluate intervention effects [7, 27].

### Conclusions and implications for clinical practice

The Schmetterling NBI Program creatively adapts techniques such as differential reinforcement, non-contingent reinforcement and escape extinction, tailoring them to individuals’ needs. Systematically measuring their effects using standardized tools like the Children’s Eating Behavior Questionnaire [15, 50]. Bridging theory and real-world impact, a key strength of the study lies in its empirical rigor and structured application of behavioral tools, which allows for measurable outcomes regarding modifying feeding behaviors in children with ASD. Furthermore, the study integrated behavioral innovation with psychometric measurement, reinforcing the credibility and replicability of our findings. By using standardized instruments and systematic observational data, the study provides quantifiable knowledge about changes in emotional overeating, satiety responsiveness, and enjoyment of food. These metrics enhance the credibility of findings and offer replicable models for future research.

Moreover, successful translation of behavioral feeding interventions from research settings into routine clinical practice remains a significant challenge. While structured program such as EAT-UP and the Schmetterling NBI demonstrate efficacy, their adoption is often hindered by health-system barriers including inadequate therapist training, high implementation costs, limited institutional support, and lack of reimbursement structures. Vivanti et al. (2018) emphasize that intervention models featuring manualized protocols, flexible delivery formats, and explicit parent-training components such as the Schmetterling Program may be more readily adopted into real-world settings. In the same vein, findings of this study carry important implications for clinical practice, particularly in the design and delivery of feeding interventions for children with ASD. The Schmetterling NBI Program demonstrates the effectiveness of non-intrusive, child-centered strategies such as exposure, positive reinforcement, and individualized intervention planning in reducing selective eating behaviors while maintaining the child’s autonomy and emotional comfort. Clinicians and behavioral therapists are encouraged to adopt these evidence-based practices to develop customized intervention plans that increase food variety, improve nutritional intake, and reduce mealtime-related distress for children with ASD and their families. Notably, the program’s emphasis on parental involvement and skill-building underscores the role of caregivers as primary agents of change, facilitating consistency, generalizability, and sustainability of intervention outcomes in clinical settings.

## Data Availability

The datasets generated and analysed during this study are available from the corresponding author upon reasonable request, subject to approval from the ethics committee and the participants’ legal guardians. Due to the small sample size and to protect the participants’ confidentiality, data will be shared only in anonymized and aggregated form where appropriate.
